# The Role of Dopamine in the Context of Aversive Stimuli with Particular Reference to Acoustically Signaled Avoidance Learning

**DOI:** 10.3389/fnins.2012.00132

**Published:** 2012-09-14

**Authors:** Anton Ilango, Jason Shumake, Wolfram Wetzel, Henning Scheich, Frank W. Ohl

**Affiliations:** ^1^Leibniz Institute for NeurobiologyMagdeburg, Germany; ^2^Institute of Biology, Otto-von-Guericke UniversityMagdeburg, Germany; ^3^Department of Psychology, The University of TexasAustin, TX, USA

**Keywords:** dopamine, aversive stimuli, avoidance learning, intracranial self-stimulation, reward and punishment, dorsal vs. ventral striatum, lateral habenula, ventral tegmental area

## Abstract

Learning from punishment is a powerful means for behavioral adaptation with high relevance for various mechanisms of self-protection. Several studies have explored the contribution of released dopamine (DA) or responses of DA neurons on reward seeking using rewards such as food, water, and sex. Phasic DA signals evoked by rewards or conditioned reward predictors are well documented, as are modulations of these signals by such parameters as reward magnitude, probability, and deviation of actually occurring from expected rewards. Less attention has been paid to DA neuron firing and DA release in response to aversive stimuli, and the prediction and avoidance of punishment. In this review, we first focus on DA changes in response to aversive stimuli as measured by microdialysis and voltammetry followed by the change in electrophysiological signatures by aversive stimuli and fearful events. We subsequently focus on the role of DA and effect of DA manipulations on signaled avoidance learning, which consists of learning the significance of a warning cue through Pavlovian associations and the execution of an instrumental avoidance response. We present a coherent framework utilizing the data on microdialysis, voltammetry, electrophysiological recording, electrical brain stimulation, and behavioral analysis. We end by outlining current gaps in the literature and proposing future directions aimed at incorporating technical and conceptual progress to understand the involvement of reward circuit on punishment based decisions.

## Introduction

According to Skinner (1938), events that strengthen or increase the likelihood of preceding responses are called positive reinforcers, and events whose *removal* strengthens preceding responses are called negative reinforcers. Based on the affective attributes that determine the reinforcing nature of the unconditioned stimulus (US), these can also be classified as appetitive and aversive reinforcers, respectively (Konorski, 1967). Decades of research have documented phasic (short latency and short duration) dopamine (DA) signals evoked by appetitive reward or conditioned reward predictors and the modification of these signals by changes in reward value (e.g., magnitude, probability, and delay) or reward omission (Schultz et al., 1997). However, the DA neuron response to aversive reinforcers as a function of punishment prediction or avoidance has received far less research attention. Here we review convergent findings, obtained utilizing microdialysis, voltammetry, electrophysiological recording, and electrical brain stimulation, indicating that DA not only plays a role in coding aversive stimuli, but also serves essential functions for the formation of behavioral learning strategies aimed at the avoidance of aversive stimuli.

## The Dopaminergic System and Aversive Stimuli

The release of DA in the context of aversive stimuli has been extensively studied using microdialysis. For example, after stressful tail-stimulation extracellular DA levels were increased in the dorsal striatum, nucleus accumbens (NAc), and medial prefrontal cortex (PFC), suggesting involvement of nigrostriatal, mesolimbic, and mesocortical DA systems (Abercrombie et al., 1989; Boutelle et al., 1990; Pei et al., 1990). Moreover, regional differences in DA release have been demonstrated within the ventral striatum in response to aversive stimuli. Prolonged administration of footshock increased extracellular DA in the NAc shell but not core (Kalivas and Duffy, 1995). Furthermore, presentation of sensory stimuli preconditioned with footshock elevated DA levels in NAc (Young et al., 1993). Pretreatment with footshock over several days decreased cocaine-induced DA elevation in mPFC but increased DA in the NAc (Sorg and Kalivas, 1991, 1993; Ungless et al., 2010). In some studies, the DA response to aversive stimuli declined with repeated stress exposure (Imperato et al., 1992). Across studies, different experimental procedures (seconds vs. minutes; 1 min sampling period vs. 10 min sampling period; brief, novel aversive stimuli vs. repeated, chronic aversive stimuli) have made it difficult to draw coherent conclusions.

While microdialysis is useful for directly measuring the localized concentration of DA within a brain region, its temporal sensitivity is limited, usually reflecting more tonic fluctuations in DA release averaged across intervals of 2–10 min. Fast scan cyclic voltammetry (FSCV), on the other hand, is an indirect measure of DA release interpreted from the electrical currents associated with the oxidation and reduction of DA but has high temporal resolution (on the order of 200 ms), which is capable of detecting phasic DA signals associated with a single learning trial. A recent study clarified the role of DA for processing appetitive and aversive reinforcers by measuring the phasic DA signal every 100 ms using FSCV in response to opposite hedonic taste stimuli (rewarding sucrose vs. aversive quinine). A strong DA increase in response to sucrose and DA decrease in response to quinine was found in the NAc and dorsolateral bed nucleus of the stria terminalis, suggesting suppression of DA in these two regions in response to aversive taste stimuli (Roitman et al., 2008; Park et al., 2012). However, a 3 s tail pinch with a soft rubber glove led to different results. A phasic DA increase was time-locked to the tail pinch in the dorsal striatum and NAc core, while an increase in the NAc shell was evident once the tail pinch was removed (Budygin et al., 2012). This suggests that the delivery and removal of aversive stimuli may trigger different DA responses in different projection regions. In addition to phasic DA transients in the NAc core time-locked to aversive physical stimuli, spontaneous DA transients have also been reported in response to aversive social confrontations, such as facing an aggressive resident followed by social defeat (Anstrom et al., 2009). The difference in phasic DA transients in the NAc shell and core in response to aversive events is consistent with a specific motivational role executed by different DA pathways (Salamone, 1994; Ikemoto and Panksepp, 1999; Salamone and Correa, 2002; Ikemoto, 2007).

On the level of single neuron activity, aversive stimuli have often been reported to inhibit phasic DA neuron firing in several species (Mirenowicz and Schultz, 1996; Schultz et al., 1997; See Table 1). However, some studies also reported increased phasic firing in response to an aversive conditioned stimulus (CS; e.g., Guarraci and Kapp, 1999). To gain further insights into such discrepant results, recent studies combined extracellular recording and unit identification by juxtacellular neurobiotin labeling (Ungless et al., 2004; Brischoux et al., 2009; Mileykovskiy and Morales, 2011). In response to aversive footshock, DA neurons from different components of the VTA (the dorsal parabrachial pigmented nucleus and the ventral paranigral nucleus) showed opposite modulation of firing, i.e., a reduction and an increase, respectively (Ungless et al., 2004; Brischoux et al., 2009). Valenti et al. (2011) further demonstrated that a single footshock inhibited most of the recorded DA neurons, but repeated footshock evoked different responses on DA neuronal population activity along the mediolateral direction, with predominant excitation on the medial side. Also, DA neurons which were inhibited by the CS signaling the arrival of aversive airpuff were located more medially in VTA and substantia nigra pars compacta (SNc) medial part as opposed to the lateral SNc DA neurons which were predominantly excited (Matsumoto and Hikosaka, 2009).

**Table 1:** **Effect of aversive stimuli on mid brain DA neurons**.

Citation	Methodological details	Aversive stimuli (US) or Aversive conditioned stimuli (CS)	Regions recorded	% Neuron response to aversive stimuli	% Neuron response to offset of aversive stimuli	Notes
Brown et al. (2009)	Extracellular recording in urethane anesthetized rats	Pinches of 15 s duration to the hindpaw	SNc	18% inhibition to aversive stimuli		Other recorded neurons did not respond to either pinch or electrical shock
		Footshocks (0.5 Hz and 2 ms duration) at 5 mA intensity were delivered for 100 trials to the hind paw		20% inhibition to aversive stimuli
Brischoux et al. (2009)	Extracellular recording and juxtacellular labeling in urethane anesthetized rats	Footshocks (20 Hz, 5 mA) and 4 s trains	VTA: dorsal parabrachial nucleus	55% of labeled were inhibited		Others did not respond to shock
			VTA: ventral paranigral nucleus			Labeled DA neurons excited by footshock was reported
Cohen et al. (2012)	Extracellular recording (Tetrode) in DAT-Cre mice identification using optical stimulation of channelrhodopsin	Air puff to the face	VTA	All optogenetically identified DA neurons were inhibited for the aversive stimuli		<50% of optogenetically identified DA neurons were excited by reward predicting CS
Coizet et al. (2006)	Extracellular recording in urethane anesthetized rats	Footshock (0.5 Hz and 2 ms duration) at 5 mA intensity were delivered for 60 trials	SNc	72% inhibited		
				12% excited
Coizet et al. (2010)	Extracellular recording in urethane anesthetized rats	Footshock (0.5 Hz and 2 ms duration) at 5 mA intensity were delivered for 100 trials	Dorsal SNc	<80% showed inhibition of firing		Inactivation of parabrachial nucleus abolished the nociceptive responses
Gao et al. (1990)	Extracellular recording in chloral hydrate anesthetized rats	Peripheral nociceptive stimulation (shock to the tail) of 1 ms and 15–20 mA	SNc	78% inhibited		Stimulation of LHb increased the inhibitory responses
				15% excited
Gao et al. (1996)	Extracellular recording in chloral hydrate anesthetized rats	Peripheral nociceptive stimulation (shock to the tail) of 1 ms and 15–20 mA	SNc	<90% inhibited		LHb neurons showed opposite patterns
Mantz et al. (1989)	Extracellular recording and antidromic identification in ketamine anesthetized rats	Tail pinch for 10 s using forceps	Ventromedial mesencephalic tegmentum	mPFC projecting neurons: 65% excited and 25% inhibited		
				NAc projecting neurons: 4% inhibited	
Matsumoto and Hikosaka (2009)	Extracellular recording in behaving monkeys	Airpuff to the face	SNc and VTA	US 45% inhibited 10.6% excited 43.6% no response CS 23% inhibited 36.8 excited 39.8% no response		Similar responses for reward omission All, aversive CS inhibited neurons were excited for the reward conditioned CS Medial VTA and medial SNc neurons are predominantly inhibited by CS predicting air puff
Mirenowicz and Schultz (1996)	Extracellular recording in behaving monkeys	CS predicting air puff to the hand	SNc, VTA, and retrorubral field	31% inhibited <14% excited		<70% excited by the CS predicting juice reward
Mileykovskiy and Morales (2011)	Extracellular recording and juxtacellular labeling in awake rats	Fear conditioning: tone paired with tail shock (0.5–1.2 mA, 60 Hz, 1 s)	VTA	In fear conditioned rats		Correlations between inhibitory response and rats which discriminates the fear CS
				Type 1 (60%): inhibited for onset of CS^+^	
				Type 2 (20%): inhibited for onset and offset of CS^+^	
				Type 3 (20%): biphasic excitatory or inhibitory responses followed by inhibitory pause
Tsai et al. (1980)	Extracellular recording in urethane anesthetized rats	Single shock stimulation to sciatic nerve (square pulse of 4–10 V intensity and 0.3 ms duration	SNc	<85% inhibited 6% excited Prolonged duration of inhibition	Rebound excitation Rebound excitation	
		Repeated footshocks (10–50 Hz)			
Ungless et al. (2004)	Extracellular recording and juxtacellular labeling in urethane anesthetized rats		VTA	83% are inhibited Others are non-responsive		
Wang and Tsien (2011)	Extracellular recording (Tetrode) in behaving mice	20 trials of fearful events (Free fall from 10 to 30 cm height and shake from 0.2–1 s) with 1–2 min inter-trial interval	VTA	Type 1 (59%): Suppression of firing in response to both events	Type 1:offset-rebound excitation	96% of the type 1 and type 2 DA neurons excited by the CS signaling reward
				Type 2 (13%): Suppression of firing in response to both events	No effect	

Mileykovskiy and Morales (2011) studied the response of VTA DA neurons to a CS paired with a tail shock US. Three types of responses from DA neurons were observed during the presentation of the aversive CS, some of which featured biphasic inhibition and excitation. But all of the response types featured an inhibitory pause in firing, the duration of which was correlated with the expression of fear.

Furthermore, inhibition of DA neuron (59%) firing evoked by fearful events such as free fall and shake was followed by offset-rebound excitation (phasic burst firing) upon their termination. Interestingly, the same DA neurons also displayed a reward prediction signal (modulated firing in response to a stimulus that is associated with later occurrence of a reward) when conditioned later with sugar pellet (Wang and Tsien, 2011). From the available evidence including recent optogenetic insights, our understanding of DA neuron response to appetitive and aversive stimuli has broadened. The vast majority of DA neurons appear excited by appetitive rewards and their predictors, and inhibited by aversive punishments and their predictors, as well as by reward omission (Tobler et al., 2003; Mileykovskiy and Morales, 2011; Wang and Tsien, 2011; Cohen et al., 2012).

## Dopamine and Avoidance Learning

Avoidance learning is the process by which an individual learns a behavioral response to avoid aversive stimuli. An important feature of avoidance learning is that it is governed by *negative* reinforcement; that is, the *absence* of a stimulus motivates behavioral change. The mechanism for exactly how the absence of something can come to serve as a reinforcer has been a puzzle for learning theorists and the focus of much behavioral research. A popular theory accounting for this phenomenon is the two-process theory of avoidance (Dinsmoor, 2001), which states that an animal first learns a Pavlovian association that a CS, such as a tone, will be followed by an aversive US, such as a shock. This Pavlovian association then becomes the basis for operant learning, in that the CS becomes aversive in its own right and thus capable of motivating an operant response. The two-process theory proposes that the CS triggers a state of fear, which the animal then acts to reduce. Thus, fear reduction becomes the ultimate mechanism for negative reinforcement learning. However, here we outline evidence for an alternative mechanism: namely, the formation of an expectation of CS-US contingency is indeed a critical prerequisite, but the *violation of aversive expectation* when the animal performs the correct avoidance response directly activates the DA reward system. Thus, the ultimate mechanism for negative reinforcement learning is isomorphic with that of positive reinforcement learning, and it is dopaminergic.

Numerous studies have found specific effects of DA manipulations on avoidance learning. Beninger et al. (1989) found that low doses of DA antagonists impaired active avoidance responses without affecting motor behavior. Depletion of DA by 6-hydroxydopamine (6-OHDA) in the SNc (Cooper et al., 1973; Jackson et al., 1977; Salamone, 1994), NAc (McCullough et al., 1993), or PFC (Sokolowski et al., 1994) impaired the development and maintenance of active avoidance strategies, usually without affecting motor responses, including escape responses. Active avoidance behavior was also disrupted by alpha-methyl-p-tyrosine injections in NAc and rescued by DA injections (Bracs et al., 1982). The D2 antagonist sulpiride inhibited avoidance learning when injected into NAc, but not when injected into PFC, amygdala, or caudate putamen (Wadenberg et al., 1990). However, other studies found that D2 antagonist injections into NAc did not impair acquisition but did reduce conditioned responding during subsequent tests, whereas D1 antagonist injections into NAc impaired conditioned responding during both acquisition and subsequent testing (Boschen et al., 2011; Wietzikoski et al., 2012).

While it seems clear from these studies that some dopaminergic target regions play a DA-dependent role in avoidance learning, it is not yet fully transparent what this role is or which DA receptors are essential for it. Extensive work in our laboratory has addressed these questions using shuttle-box avoidance learning, either conditioned by a frequency-modulated (FM) tone or by a GO-NO GO discrimination paradigm using rising and falling FM tones, the processing of which depends on auditory cortex (Wetzel et al., 1998, 2008; Ohl et al., 1999). Microdialysis in auditory cortex and medial PFC showed that DA release in both structures reaches a peak during the first few trials of successful avoidance (Stark et al., 2001, 2008). The consequences of this initial DA release were clarified by subsequent reversal learning experiments, in which a consolidated GO response to two oppositely modulated FM tones was challenged by switching the requirement for one of the FM tones to a NO GO response (Stark et al., 2004). This resulted in an initial breakdown in avoidance responding to chance levels for all animals. However, some animals showed improvement in discrimination learning over subsequent days, and only these animals showed strong DA release in mPFC. This suggests an association between mPFC DA and the discovery of correct discrimination contingencies, and a facilitative or perhaps even causal role for DA in the formation of successful go vs. no go discrimination.

Neuronal activity in auditory cortex is known to be influenced by dopaminergic inputs (e.g., Bao et al., 2001) compatible with the anatomical connectivity from the VTA to the auditory cortex (e.g., Budinger et al., 2008). To investigate the role of specific DA receptors in auditory discrimination learning, a variety of DA agonists and antagonists were administered bilaterally to the auditory cortex both before and after training (Tischmeyer et al., 2003; Schicknick et al., 2008, 2012). The chief conclusion from these experiments was that only drugs affecting D1/D5 receptors are capable of depressing or enhancing discrimination learning. The most interesting effect was that the D1 agonist SKF 38393 injected before training did not influence acquisition during the training session but did lead to improved retrieval the next day. This effect was blocked by concurrent application of rapamycin, a specific inhibitor of the protein kinase mTOR implicated in the control of synaptic protein synthesis and relevant for memory consolidation in discriminative avoidance learning (Kraus et al., 2002). Taken together, these experiments suggest that DA release in auditory cortex is necessary for the FM tone conditioned avoidance response, and may enhance memory consolidation via a D1-receptor-mediated pathway.

While the administration of pharmacological agents is useful for elucidating specific receptor pathways, this approach is limited in that it alters tonic neuromodulation over a prolonged period of time without informing, and perhaps even interfering with, the role of dynamic neuromodulation, i.e., the up-and-down fluctuations in neuromodulators over very short time scales. Based on the evidence outlined in the previous sections, such phasic changes in the DA signal may be especially relevant to incentivized learning. Specifically, DA neurons are known to respond to the omission of an expected appetitive stimulus with a momentary cessation in firing. We theorized that DA neurons would greet the omission of an expected aversive stimulus in a symmetrical manner, namely, with a transient burst in firing. Signaled active avoidance learning inherently leads to such a negative expectation (e.g., shock will follow tone) as well as its subsequent violation (e.g., shock does not follow tone if hurdle is promptly crossed). Could DA involvement in avoidance learning be specific to the trials when the expectation of shock is violated, that is, when the animal first performs a successful avoidance response? Could a pronounced DA increase at this critical moment be responsible for reinforcing the avoidance response?

**Figure 1:**
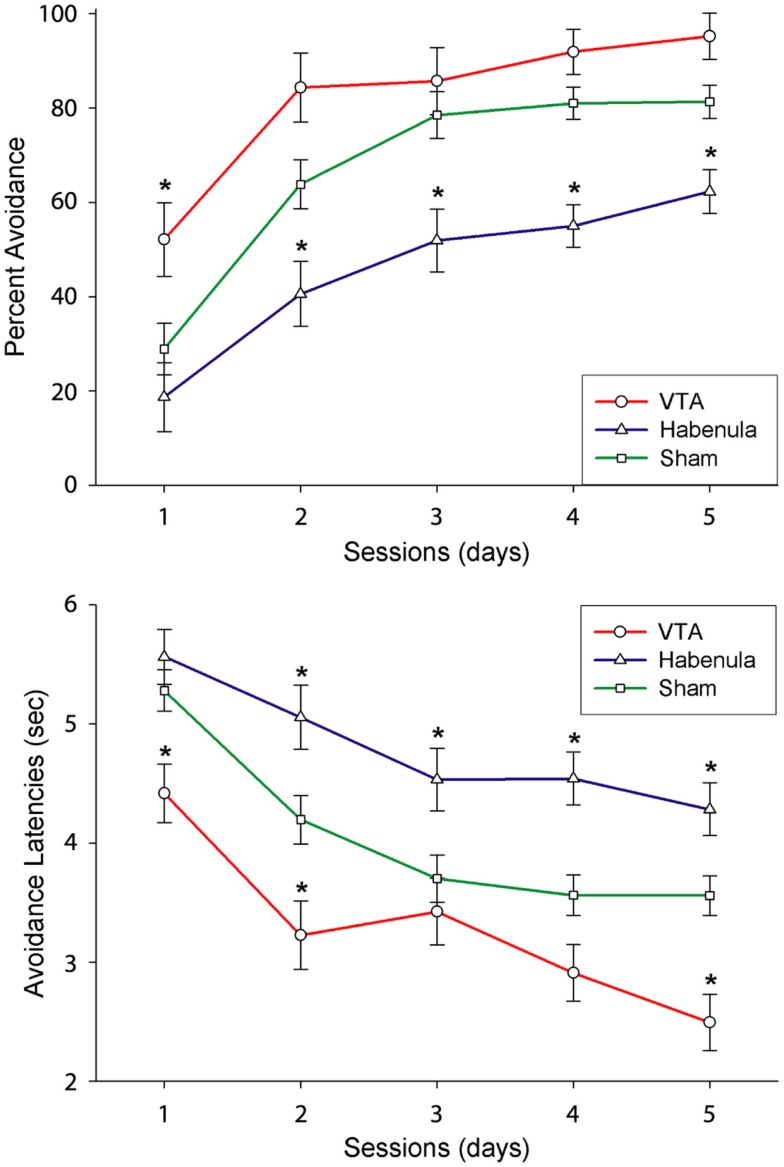
**Effect of VTA vs. LHb stimulation on the acquisition of avoidance**. Upper and lower panels indicate the Mean and SEs of successful avoidance trials and avoidance latency. Asterisks indicate significant differences between stimulated vs. control group (Modified from “Shumake et al., 2010”).

If so, a transient disruption of DA transmission following the initial trials of successful avoidance responding (when the animal is pleasantly surprised by the absence of shock) should disrupt learning. On the other hand, an equivalent manipulation following later trials after the avoidance response is well learned (when the animal fully expects that its behavior will lead to the absence of shock) should have no effect. Electrical stimulation of the lateral habenula (LHb), which results in transient, widespread inhibition of DA neurons in rodents and primates (Christoph et al., 1986; Ji and Shepard, 2007; Matsumoto and Hikosaka, 2007), was used to test this hypothesis (Shumake et al., 2010). Specifically, we implanted the LHb with a stimulation electrode and delivered brief electrical stimulation whenever the animal performed a correct avoidance response, i.e., when the initial avoidance of foot shock was hypothesized to trigger an intrinsic reward signal. As predicted, LHb stimulation initiated early in training impaired learning, but LHb stimulation initiated late in training had no effect (Shumake et al., 2010; Figure 1). These findings suggest a vital role for phasic DA signaling in the successful acquisition of active avoidance behavior. What is not yet clear is whether the presumed phasic increases in DA add up to the tonic increases in forebrain DA levels previously observed (Stark et al., 1999, 2000; Giorgi et al., 2003), or whether phasic and tonic DA signals convey differential information in the context of avoidance learning.

## Brain Stimulation Reward and Avoidance Learning

Since James Olds discovered intracranial self-stimulation (ICSS; Olds and Milner, 1954; Olds, 1958), several ICSS-supporting regions have been characterized. The majority of these regions lie along DA projections, such that robust ICSS can be evoked from the VTA, substantia nigra, and lateral hypothalamus. Moreover, extracellular DA elevation is necessary to maintain ICSS (Fibiger et al., 1987; Fiorino et al., 1993; Owesson-White et al., 2008). Over the years, the effects of brain stimulation reward (BSR) were studied in learning and memory experiments, and it was found that BSR applied as experimenter-delivered stimulation or self-stimulation by the animal facilitated avoidance learning (Mondadori et al., 1976; Huston et al., 1977; Destrade and Jaffard, 1978; Segura-Torres et al., 1988, 1991, 2010; Huston and Oitzl, 1989; Aldavert-Vera et al., 1997; Ruiz-Medina et al., 2008). These results show that BSR given before or after training led to improvement of avoidance learning by improving the learning efficiency. However, correct avoidance responding is reinforced not only by terminating the aversive warning signal, i.e., relief from fear, but also by producing a safety signal, i.e., response-generated feedback stimuli signaling safety (Cicala and Owen, 1976; Dinsmoor, 1977; Masterson et al., 1978). Concerning the aversive component, the potential for enhancing the strength of reinforcement, e.g., by increasing shock intensity, is rather limited. Concerning the appetitive component, however, it is possible to enhance the magnitude of reinforcement by using additional feedback stimuli, e.g., sensory cues contingent to avoidance (Morris, 1975; Cicala and Owen, 1976), access to a safe place (Modaresi, 1975; Baron et al., 1977), or handling during the inter-trial interval (Wahlsten and Sharp, 1969).

These data support the view that any stimuli negatively correlated with shock, whether exteroceptive (presented by the experimenter) or interoceptive (presented by the subject’s own behavior), are inherently rewarding (Dinsmoor, 2001). Compatible with this idea, recent MRI studies in humans have suggested that activity in the medial orbitofrontal cortex, a reinforcement evaluating area, reflects an intrinsic reward signal that serves to reinforce avoidance behavior (Kim et al., 2006). Thus, we can assume that in aversively motivated learning, avoidance learning responses come under the control of positive incentives. Earlier investigations on appetitive-aversive interactions have shown that appetitive training appears to facilitate subsequent aversive conditioning (Dickinson, 1976; Dickinson and Pearce, 1977) and that operant behavior is enhanced by using concurrent schedules of positive and negative reinforcement (Kelleher and Cook, 1959; Olds and Olds, 1962). Moreover, a few studies reported the facilitation of discrete-trial avoidance (Stein, 1965; Castro-Alamancos and Borrell, 1992) and Sidman avoidance (in which shock is not signaled but rather occurs at fixed intervals unless the animal performs the operant response; Margules and Stein, 1968; Carder, 1970) by *non-contingent* rewarding brain stimulation, an effect resembling the action of stimulant drugs like amphetamine on self-stimulation and avoidance performance.

These results support the idea that the brain reward system facilitates operant behavior, whether positively or negatively reinforced. Not tested, however, was the effect of BSR given *contingently* to a correct response, i.e., exactly during the time-point when the response-generated safety signal occurs. Thus, in our studies we used the shuttle-box two-way avoidance paradigm to provide a way to combine BSR with footshock negative reinforcement to drive the same learned operant behavior. We found that this reinforcer combination potentiated the speed of acquisition, led to superior (nearly 100% correct) performance and delayed extinction, as compared to either reinforcer alone (Ilango et al., 2010, 2011; Shumake et al., 2010). These findings demonstrate that adding intrinsic reward (by stimulating dopaminergic structures) to the relief from punishment results in maximum avoidance performance, supporting the view that brain reward circuits serve as a common neural substrate for both appetitively and aversively motivated behavior.

## Perspectives

In conclusion, several lines of evidence strongly argue in favor of the involvement of reward circuitry for the processing of aversive stimuli, especially to encode their predictors and to form an instrumental strategy to avoid them (e.g., Brischoux et al., 2009; Matsumoto and Hikosaka, 2009; Bromberg-Martin et al., 2010; Ilango et al., 2010, 2011; Budygin et al., 2012). Specifically, the neurotransmitter DA is involved in neuronal and behavioral responses to cues predicting reward (approach) or punishment (avoidance), both of which are vital for adaptive behavior. Electrophysiological signatures obtained from VTA DA neurons have begun to reveal their convergent encoding strategy for mediating both appetitive and aversive learning (Kim et al., 2012). Furthermore, VTA BSR can be integrated into avoidance learning tasks to investigate the nature of reinforcer interaction, and to understand the similarity between affective states associated with absence of predicted appetitive stimuli (frustration) and predicted aversive stimuli (fear) vs. absence of predicted aversive stimuli (relief) and predicted appetitive stimuli (hope; Seymour et al., 2007; Ilango et al., 2010).

Further progress in understanding the neuronal basis of affective behaviors will rely on both technical and conceptual progress. On the technical side, optogenetic approaches will allow triggering temporally precise events in specific cell types. For example, driving DA neurons in VTA by channelrhodopsin has already been demonstrated to support vigorous intracranial self-stimulation and place preference (Tsai et al., 2009; Witten et al., 2011). Such approaches could be extended to clarify the respective roles of several cell populations in different behaviors.

On the conceptual side, behavioral paradigms that allow the assessment of DA-related neuronal signatures in flexible scenarios will be important. For example, deeper insight into the role of DA with respect to the dissociation between (1) the association of specific behavioral meaning to stimuli and (2) the organization of appropriate behaviors can be expected from comparison of Pavlovian and instrumental paradigms. Also, discriminative avoidance learning tasks can be used to investigate how the same DA neuron responds to a CS+ in a hit vs. a miss trial or to a CS- in false-alarm vs. a correct-rejection trial, thereby allowing assessment of which factors govern the recruitment of excitatory and inhibitory contributions to neuronal and behavioral responses.

## Conflict of Interest Statement

The authors declare that the research was conducted in the absence of any commercial or financial relationships that could be construed as a potential conflict of interest.
